# Mechanisms for the age‐related increase in fatigability of the elbow flexor muscles in healthy adults

**DOI:** 10.14814/phy2.70581

**Published:** 2025-10-03

**Authors:** Blaine E. Arney, Andrew Kuplic, Mitchell D. Adam, Christopher W. Sundberg, Sandra K. Hunter

**Affiliations:** ^1^ Department of Kinesiology Pennsylvania State University Altoona Altoona Pennsylvania USA; ^2^ Department of Physical Therapy, Exercise Science Program Marquette University Milwaukee Wisconsin USA; ^3^ School of Kinesiology University of Michigan Ann Arbor Michigan USA

**Keywords:** aging, contractile properties, muscle fatigue, sex differences, transcranial magnetic stimulation

## Abstract

The mechanisms for the age‐related increase in fatigability of the elbow flexor muscles during dynamic contractions are unknown. The purpose of this study was to determine the contribution of supraspinal and contractile mechanisms for the age‐related increase in fatigability of the elbow flexor muscles. Twenty‐eight young (23.2 ± 2.6 years; 14 females) and 32 older adults (72.6 ± 5.6 years; 15 females) performed 80 maximal velocity contractions at 20% of maximal isometric strength. Voluntary activation and muscle contractile properties were assessed before and immediately following the fatiguing exercise using transcranial magnetic stimulation (TMS) and electrical stimulation. Fatigability (% reduction in power) was greater in older (49.2%) than in young adults (37.6%; *p* = 0.01) with no differences between males and females (*p* = 0.36). Reductions in voluntary activation following exercise did not differ between young and older adults (*p* = 0.96). Reductions in peak rates of torque relaxation induced by TMS were greater in older (44.4%) than in young adults (22.8%; *p* < 0.001) and were associated with fatigability (*r* = 0.51, *p* < 0.001). These data suggest that the age‐related increase in fatigability of the elbow flexors is due primarily to mechanisms that impair muscle contractile function.

## 1 INTRODUCTION

Human aging is accompanied by changes within the neuromuscular system which lead to reductions in muscular strength, contraction velocity, and power, and increased fatigability of limb muscles during repeated dynamic contractions (Alcazar et al., 2020; Hunter et al., 2016; Paris et al., 2022; Wrucke et al., 2024). Performance fatigability of limb muscles is an acute activity‐induced reduction in force and power, and hereafter is referred to as fatigability (Enoka & Duchateau, 2016; Hunter, 2018). Fatigability of limb muscles is greater for older adults (>65 years) compared with young adults when performing dynamic contractions at high velocities in both the upper limb (Senefeld et al., 2017) and lower limb muscles [e.g., (Christie et al., 2011; Dalton et al., 2015; McNeil & Rice, 2007; Paris et al., 2022; Senefeld et al., 2017; Sundberg, Kuplic, et al., 2018)]. Age‐related losses of muscle power of the lower extremity are associated with reduced performance of functional activities (e.g., stair climbing, rising from a chair, and mobility) (Bean et al., 2008; Foldvari et al., 2000; Reid & Fielding, 2012; Wrucke et al., 2024) and can be further exacerbated by increased fatigability of limb muscles, which is independently associated with reduced physical function in older adults (Senefeld et al., 2017).

Fatigability of the lower and upper limb during high‐velocity contractions is pronounced in older adults even after 4–6 min of repeated contractions (Knee Extensors = ~25%–40%; Elbow Flexors = ~20%) (Senefeld et al., 2017; Sundberg, Kuplic, et al., 2018). Additionally, we have shown that age differences in fatigability in the knee extensor muscles are over twice that of the elbow flexor muscles (Senefeld et al., 2017). This age‐related difference in the magnitude of fatigability between the knee extensor and elbow flexor muscles may be due, in part, to varying activity patterns between the upper and lower limb leading to a greater preservation of skeletal muscle mass, velocity, and torque production in the upper limb muscles with aging (Frontera et al., 2000; Janssen et al., 2000; Kern et al., 2001; Naruse et al., 2023). However, little is known about the mechanisms of the age‐related increase in fatigability of upper limb muscles among older adults during high‐velocity contractions.

In the lower limb muscles, the increased fatigability of older adults during high‐velocity dynamic contractions is primarily muscular in origin (Baudry et al., 2007; Dalton et al., 2010, 2012; Paris et al., 2022; Sundberg, Kuplic, et al., 2018). For example, it was observed that the age‐related increase in fatigability of the knee extensor muscles during moderate‐ to high‐velocity contractions was closely associated with the reduction in the electrically evoked peak twitch torque and indices of slowed contractile properties, including reduced rates of torque development and prolonged half‐relaxation times (Sundberg, Kuplic, et al., 2018). Additionally, transcranial magnetic stimulation (TMS) coupled with femoral nerve stimulation revealed that the greater age‐related fatigability of the knee extensor muscles was not due to age differences in neural drive or neuromuscular propagation (Sundberg, Kuplic, et al., 2018). Whether the increased fatigability of the upper limb muscles during high‐velocity contractions in older males and females is primarily due to mechanisms within the nervous system or in the muscle is not known. Given the complex neural connections of the upper limb, and the larger supraspinal fatigue observed with aging during isometric fatiguing contractions performed with the elbow flexor muscles (Hunter et al., 2008; Yoon et al., 2012), reductions in neural drive may be more involved in the age‐related increase in fatigability of the upper limb muscles. Moreover, it is unknown whether the mechanisms for the age‐related increase in fatigability of the upper extremity differ between males and females. Unlike the findings from the knee extensor muscles (Sundberg, Kuplic, et al., 2018), older males demonstrated greater reductions in contraction velocity compared with older females during high‐velocity exercise of the elbow flexor muscles (Senefeld et al., 2017), suggesting that there may be sex differences in the mechanisms of fatigability for the upper extremity.

Thus, the purpose of this study was to determine the mechanisms associated with the age‐related increase in fatigability during high‐velocity contractions of the elbow flexor muscles and to test for sex‐based differences in the mechanisms of fatigability with age. We hypothesized that older adults will be more fatigable than young adults during a high‐velocity fatiguing exercise of the elbow flexor muscles due to mechanisms primarily within the muscle, with a small contribution from impairments in the ability of the nervous system to activate the muscle. We also hypothesized that fatigability would be greater in older males than older females primarily due to muscular mechanisms.

## 2 METHODS

### 2.1 Participants

Twenty‐eight young adults (23.2 ± 2.6 years; range = 19.5–29.1 years; 14 males and 14 females) and 32 older adults (72.6 ± 5.6 years; range = 61.9–83.8 years; 17 males and 15 females) participated in the study. All participants were healthy, community‐dwelling males and females with no known neurological disease or contraindications to exercise. Participants were screened and excluded for the use of medication affecting the central nervous system and hormonal status (e.g., hormone‐replacement therapy). Prior to the experiment, each participant provided written informed consent. The protocol was approved by the Marquette University Institutional Review Board and was conducted according to the Declaration of Helsinki. Physical characteristics and physical activity levels of the participants are presented in Table 1.

**TABLE 1:** Anthropometrics and physical activity levels for the young and older males and females.

Variable	Young		Older	
	Male (14)	Female (14)	Male (17)	Female (15)
Age, yr*	24.0 ± 2.2	22.3 ± 2.8	71.8 ± 5.0	73.4 ± 6.4
Height, m* ^,^ ^#^	1.79 ± 0.08	1.65 ± 0.07	1.76 ± 0.09	1.59 ± 0.05
Weight, kg^#^	81.9 ± 12.1	67.5 ± 12.7	82.0 ± 11.5	64.5 ± 10.3
Body mass index, kg/m^2^	25.4 ± 2.6	24.7 ± 3.7	26.3 ± 2.7	25.4 ± 3.9
Body fat, %* ^,^ ^#^	20.0 ± 4.9	33.1 ± 6.8	28.6 ± 5.2	38.2 ± 6.3
Physical activity, steps/day	8391 ± 3276 (11)	9483 ± 3849 (10)	9063 ± 3987 (15)	8771 ± 4083 (14)

*Note*: Values are reported as mean ± SD. The sample sizes (*n*) for each cohort and certain variables are reported in parentheses.

* *p* < 0.05, significant effect of age.

^#^ *p <* 0.05, significant effect of sex.

### 2.2 Experimental setup

Each participant was seated upright in an adjustable chair (Biodex Medical System 4, Shirley, NY). The shoulder was flexed to 50° in the sagittal plane (with 0° considered to be in line with the torso), and the elbow rested comfortably on a padded support. The forearm was placed in a fully supinated position within an orthosis (Orthomerica, Newport Beach, CA), which was attached to the lever arm of a Biodex dynamometer (Biodex Medical System 4, Shirley, NY). The axis of rotation of the dynamometer was aligned to the anatomical axis of the elbow of the participant. Each participant was secured across both shoulders and the waist with padded straps to minimize extraneous movements during contractions. For isometric contractions, the elbow joint was flexed to 90°. Concentric contractions were performed over an 80° range of motion (170°–90°). Recordings of muscle torque, velocity, and position from the Biodex dynamometer were digitized by a Power 1401 analog‐to‐digital converter and Spike 2 software (Cambridge Electronics Design, Cambridge, UK) with a sampling rate of 1000 Hz.

#### 2.2.1 Electrical recordings

EMG signals were recorded with bipolar silver chloride circular (8 mm diameter) surface electrodes that were placed over the biceps brachii (BB), brachioradialis (BR), and triceps brachii (TB) according to standard recommendations (Hermens et al., 2000). For BB, the electrodes were placed between the medial acromion and fossa cubit, 1/3 from the fossa cubit. For the BR, the electrodes were placed on the muscle belly ~4 cm distally from the lateral epicondyle. Reference electrodes were placed on the acromion. For the TB, the electrodes were placed on the long head midway between the posterior crista of the acromion and the olecranon at 2 finger widths medial to the line. The EMG signals were amplified (100×), band‐pass filtered (13–1000 Hz; Coulbourn Instruments, Allentown, PA), and sampled at 2000 Hz via a Power 1401 analog‐to‐digital converter and stored online using Spike 2 software (Cambridge Electronics Design, Cambridge, UK).

#### 2.2.2 Stimulations

Participants were stimulated at the brachial plexus and over the BB muscle with electrical stimulation, and at the motor cortex with TMS.

#### 2.2.3 Brachial plexus stimulation

The brachial plexus was electrically stimulated to produce a maximal compound muscle action potential (M_max_) of the BB, BR, and TB muscles at rest. Single stimuli (400 V and 100 μs duration) were delivered to the brachial plexus using a constant‐current stimulator (model DS7AH, Digitimer, Hertfordshire, UK). A cathode was placed in the supraclavicular fossa and an anode over the acromion. The stimulation intensity was determined by increasing the current until the peak‐to‐peak M‐wave amplitude plateaued and was then increased by 20% to ensure a maximal electrical response. The stimulation intensity ranged between 50 and 300 mA and once the intensity was determined, this level of stimulation was used for the remainder of the protocol.

#### 2.2.4 Muscle stimulation

The BB muscle was directly stimulated to produce a maximal twitch of the elbow flexor muscles. Paired (400 V, 100 μs duration and 100 Hz) stimuli (doublet) were delivered by a constant‐current stimulator (DS7AH, Digitimer) through custom‐made electrodes (2.0 × 4.5 cm) attached to the skin overlying the BB muscle. The cathode was placed directly over the muscle belly, midway between the anterior edge of the deltoid and the elbow crease. The anode was placed over the distal biceps tendon. The optimal intensity for doublet stimulations was determined by increasing the stimulator intensity until a plateau occurred in the twitch torque amplitude. This intensity was then increased by 20% to ensure maximal twitch responses and was used for the remainder of the experimental session.

#### 2.2.5 Motor cortex stimulation

TMS was delivered via a round coil (13.5 cm outside diameter; Magstim 200, Magstim, Whitland, UK) over the vertex of the motor cortex to evoke motor‐evoked potentials (MEP) in the BB, BR, and TB muscles during isometric elbow flexion contractions. The vertex of the motor cortex was identified and marked to ensure repeatability of coil placement throughout the protocol. The direction of current flow in the coil preferentially activated the motor cortex in the hemisphere opposite of the limb studied. Single stimuli were delivered over the motor cortex at an intensity that produced a large MEP in the agonist BB muscle (minimum amplitude of 50% of M_max_) but only a small MEP in the antagonist TB muscle (<15% M_max_) during a brief contraction of the elbow flexor muscles at 50% MVIC (Todd et al., 2004).

### 2.3 Experimental protocol

**FIGURE 1:**
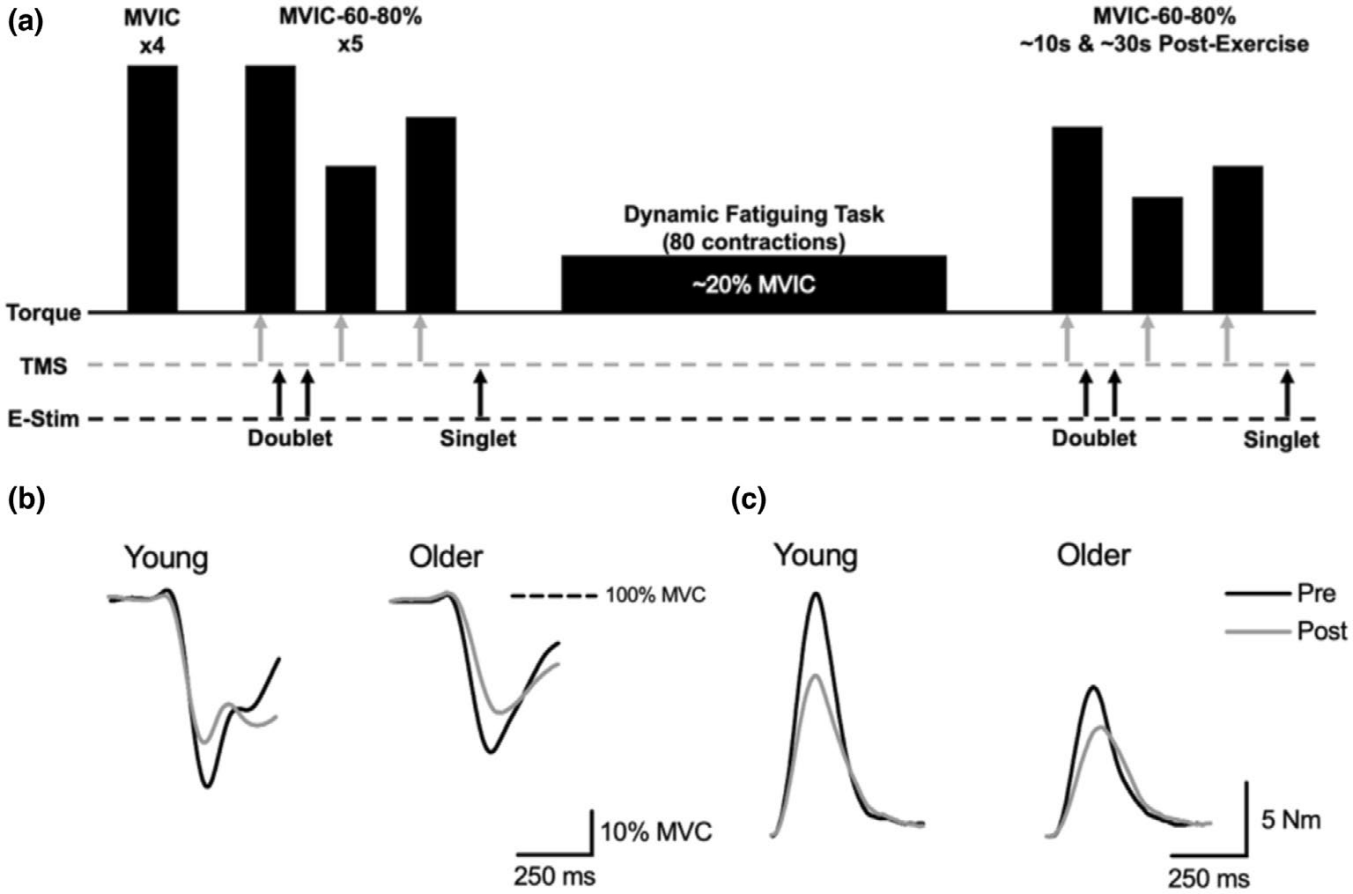
Experimental protocol. (a) shows a schematic of the experimental protocol. Following participant setup and determination of stimulator placement and supramaximal stimulator intensities, participants performed four maximal voluntary isometric contractions (MVIC) of the elbow flexor muscles. Participants then performed five sets of contractions with transcranial magnetic stimulation (TMS) at the force plateau of the MVIC and contractions at 60% and 80% of MVIC torque (MVIC‐60‐80%). Concurrently, doublet muscle electrical stimulation (E‐stim) was applied during the MVIC and at rest following the MVIC. A single brachial plexus E‐stim (singlet) was applied at rest following the three contractions. Participants then performed a 4‐min dynamic, high velocity fatiguing task (80 contractions) with a load of 20% MVIC of the elbow flexor muscles. Immediately following the dynamic fatiguing task, MVIC‐60‐80% contractions were repeated with corresponding TMS and E‐stim at ~10 and ~30 s post‐exercise. (b) and (c) displays representative data from superimposed TMS at the force plateau of the MVIC (b) and potentiated resting twitches (c) before (Pre = black line) and immediately after (Post = gray line) the fatiguing exercise task for both a young and older male.

Each participant attended one experimental session that consisted of brief maximal and submaximal isometric contractions and evoked contractions with electrical stimulation and TMS before and after a 4‐min dynamic fatiguing task with the elbow flexor muscles. A representation of the experimental protocol is provided in Figure 1. The experimental session began with measurements of the electrically evoked M‐wave and maximal twitch torque, followed by performance of maximal voluntary isometric contractions (MVIC) of the elbow flexor muscles. Four sets of brief MVICs (2–3 s for each contraction) of the elbow flexor muscles were performed and separated by 2 min of rest to minimize fatigue. Two sets of MVICs of the elbow extensor muscles were then performed that were separated by 2 min of rest in order to normalize the TB EMG activity measured during the fatiguing contractions. Strong verbal encouragement and visual feedback were provided during each maximal effort.

#### 2.3.1 Dynamic fatiguing task

A 4‐min dynamic fatiguing task was performed with the elbow flexor muscles in the Biodex set up that involved lifting a load equal to 20% of MVIC torque. Prior to the start of the fatiguing task, each participant was familiarized with the performance requirements of the contractions and were verbally encouraged to contract as hard and fast as possible. One maximal velocity contraction was performed every 3 s over the 4‐min task for a total of 80 maximal isotonic contractions. Each participant contracted through an 80° range of motion, and the limb was passively returned to the start position within the 3 s between contractions. The fatiguing task was terminated after 4 min, and the arm was returned to 90° of flexion.

#### 2.3.2 Voluntary activation and contractile properties

Voluntary activation and contractile properties of the muscle were assessed prior to the fatiguing task and in recovery at 10 s and 30 s after termination of the fatiguing task. Voluntary activation was assessed during MVICs of the elbow flexor muscles with TMS before and after the fatiguing task. M‐wave and contractile properties were also assessed during single and doublet stimulations at rest following each MVIC contraction, respectively. Prior to the fatiguing task, five measurements of voluntary activation and contractile properties were recorded with 2.5 min of rest between each set of contractions.

### 2.4 Data analysis

#### 2.4.1 Mechanical power output and range of motion

Mechanical power output (W) was calculated as the product of the measured torque (N⋅m) and angular velocity (rad/sec) and was averaged over the concentric portion of the maximal velocity contractions. Power at the beginning of the dynamic exercise task is presented as the highest average power output of five consecutive contractions within the first 10 contractions. End‐exercise power output is presented as the average power output over the last five contractions of the dynamic exercise. The range of motion at the beginning and end of the dynamic exercise was determined as the average range of motion throughout the same maximal velocity contractions used for beginning and end‐exercise power output.

#### 2.4.2 Voluntary activation

Voluntary activation was assessed using the twitch interpolation technique with TMS (Todd et al., 2003, 2016). The torque following TMS during the MVIC (superimposed twitch) was expressed as a fraction of the estimated torque of the response evoked at rest (resting twitch). The estimation of the resting twitch was obtained for each participant by linear regression analysis of the torque of the superimposed twitch at MVIC and subsequent torque production during sets of submaximal contractions of 60% and 80% of MVIC. The resting twitch was estimated rather than measured directly due to motor cortical and spinal cord excitability increasing with activity (Thompson et al., 1991). The amplitude of the estimated resting twitch, however, can be determined from three data points with contractions above 50% MVIC (Todd et al., 2003). The estimated resting twitch was used to calculate voluntary activation through the following formula (Todd et al., 2003):

(1) $$\begin{array}{c} \text{Voluntary activation}=\\ \left(1-\text{superimposed twitch}/\text{estimated resting twitch}\right)\times 100 \end{array}$$

A majority of the regression measurements taken prior to the fatiguing task were linear (*R* ^*2*^ > 0.80), although the relationship was not linear for several participants following the fatiguing task, as seen before after a fatiguing task (Hunter et al., 2008; Sundberg, Kuplic, et al., 2018). For this reason, these estimates of voluntary activation were excluded from the statistical analysis (7 young and 14 older participants). Thus, in addition to the estimated resting twitches following the fatiguing exercise, voluntary activation was also calculated with the following formula: (Gandevia, 2001; Hunter et al., 2008; Sundberg, Kuplic, et al., 2018):

(2) $$\begin{array}{c} \text{Voluntary activation}=\text{superimposed twitch}/\\ \left(\text{MVIC}+\text{superimposed twitch}\right)\times 100 \end{array}$$

#### 2.4.3 Contractile properties

Contractile properties of the muscle stimulation were assessed at rest before and following the fatiguing task as done previously (Hunter et al., 2008; Sundberg, Kuplic, et al., 2018). The peak amplitude of the twitch was measured as an index of the force‐generating capacity of the muscle. Peak rate of twitch torque development was quantified with the derivative of the torque signal as the highest rate of torque increase over a 10‐ms interval. The half relaxation time was determined as the time (ms) it took for the torque signal to reach half of the peak torque amplitude during relaxation. Lastly, when the TMS stimulation was delivered to the motor cortex during MVICs, a brief period of involuntary relaxation occurred because of the withdrawal of descending neural drive. The TMS‐induced normalized peak relaxation rates during the involuntary relaxation period were quantified with the derivative of the torque signal as the greatest rate of torque decrease over a 10‐ms interval.

### 2.5 Statistical analysis

Data are reported as means ± standard deviation (SD) in the text, tables, and figures. Two‐way analyses of variance (ANOVAs) were conducted using age and sex as independent variables for the following variables: physical characteristics; physical activity; baseline and relative reductions in mechanical measures including power output, range of motion and MVIC torque as a result of the fatiguing exercise; baseline and relative changes in mechanistic measures including voluntary activation, M‐wave, MEP, peak twitch amplitude, half relaxation time, peak rates of torque development and TMS induced muscle relaxation as a result of the fatiguing exercise. Separate 3‐way mixed ANOVAs using the independent variables of time, age, and sex were used to compare changes in the same mechanical and mechanistic measures. For all ANOVAs, only statistically significant interactions are reported in the text. Effect sizes are reported as partial eta squared ($\eta_{\rho }^{2}$) and are defined as the following: Small effect = 0.01, medium effect = 0.06, and large effect = 0.14 (Richardson, 2011). Significant interactions were further assessed with multiple pairwise comparisons using a Bonferroni correction. Pearson product–moment correlation analyses were conducted on fatigability (% reduction in power) and mechanistic measures to identify the mechanisms of fatigability.

Analysis of normality of the data and homogeneity of variance of the data were performed using the Shapiro–Wilk and Levene's tests, respectively. The presence of outliers was assessed using box plots. If assumptions of normal distribution and/or homogeneity of variance were violated, nonparametric Mann–Whitney *U* tests were performed instead of ANOVAs comparing the dependent variable across age and sex. Bonferroni corrections were used to correct for multiple comparisons, and the adjusted *p* values are reported. Significance was determined at *p* < 0.05, and all analyses were performed with SPSS Statistics (version 28; IBM, Chicago, IL).

## 3 RESULTS

### 3.1 Power output

**FIGURE 2:**
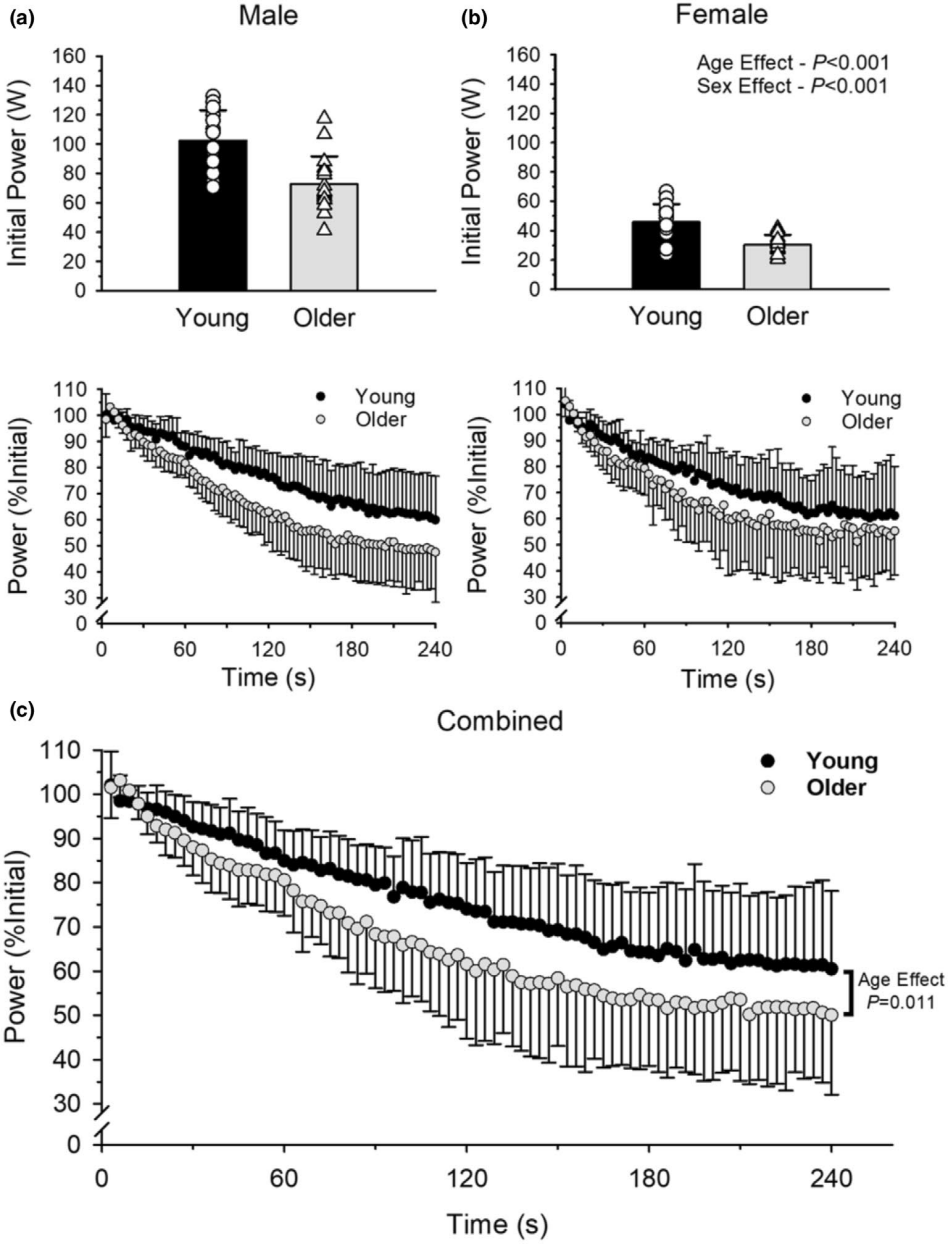
Power output during the dynamic, fatiguing exercise. (a) and (b) show mean, absolute initial power output and contraction‐by‐contraction power output (%Initial) for the dynamic exercise for the young and older adult males (a) and females (b). Due to a similar relative reduction in power between males and females (*p* = 0.356), we combined males and females (c) and displayed contraction‐by‐contraction power output (%Initial) for the separate age groups (young vs. older). Older adults experience a greater relative reduction in power compared to young (*p* = 0.011). Values are mean ± SD with individual data points (young = open circles; older = open triangles).

Power outputs at the beginning and end of the dynamic fatiguing exercise are presented in Table 2. At the beginning of the dynamic exercise, power was 40% higher in young (74.3 ± 33.3 W) compared with the older adults (53.1 ± 25.8 W; age effect, *p* < 0.001, $\eta_{\rho }^{2}$ = 0.356; Figure 2). Initial power output of males (86.3 ± 24.4 W) was 127% higher than females (38.0 ± 12.4 W; sex effect, *p* < 0.001, $\eta_{\rho }^{2}$ = 0.727) for both age groups. Young males were 123% more powerful than the young females (*p* < 0.001) and older males 139% more powerful than old females (*p* < 0.001).

**TABLE 2:** Mechanical outputs and range of motion at the beginning and end of the dynamic, fatiguing exercise.

Variable	Young		Older	
	Male (14)	Female (14)	Male (17)	Female (15)
Power (W)^♦^				
Beginning* ^,^ ^#^	102.6 ± 20.5	46.0 ± 12.2	73.0 ± 18.8	30.6 ± 6.7
End* ^,^ ^#^	64.4 ± 24.9	29.3 ± 11.3	36.3 ± 18.5	16.8 ± 6.3
Velocity (°/s)^♦^				
Beginning* ^,^ ^#^	184.3 ± 9.4	143.5 ± 17.1	145.9 ± 15.2	117.8 ± 13.4
End* ^,^ ^#^	148.6 ± 29.4	116.6 ± 25.5	104.6 ± 28.7	87.5 ± 20.7
Torque (N·m)^♦^				
Beginning* ^,^ ^#^	34.4 ± 4.9	20.0 ± 3.2	30.1 ± 5.2	17.0 ± 2.4
End* ^,^ ^#^	27.4 ± 5.11	15.9 ± 3.1	22.0 ± 5.6	13.1 ± 2.1
Range of Motion (°)^♦^				
Beginning	80.2 ± 0.7	79.7 ± 1.5	80.7 ± 0.4	79.8 ± 1.1
End	63.7 ± 5.3	63.2 ± 1.4	64.3 ± 6.9	65.8 ± 6.2

*Note*: Values are reported as mean ± SD.

^♦^ *p* < 0.05, significant effect of time.

* *p* < 0.05, significant effect of age.

^#^ *p <* 0.05, significant effect of sex.

*Fatigability* (% reduction in power) during the dynamic exercise was greater in older adults (49.2 ± 15.0%) than young adults (37.6 ± 18.2%; age effect, *p* = 0.011, $\eta_{\rho }^{2}$ = 0.111; Figure 2). Fatigability of the elbow flexor muscles did not differ between males (46.0 ± 17.0%) and females (41.5 ± 17.9%; sex effect, *p* = 0.356, $\eta_{\rho }^{2}$ = 0.015) and there was no age*sex interaction (*p* = 0.567, $\eta_{\rho }^{2}$ = 0.006).

### 3.2 Range of motion

Range of motion at the beginning and end of the dynamic exercise is presented in Table 2. All groups showed a reduction in range of motion (time effect, *p =* 0.001, $\eta_{\rho }^{2}$ = 0.904) of ~16 degrees during the fatiguing exercise with no effect of age (*p =* 0.375; $\eta_{\rho }^{2}$ = 0.014), sex (*p =* 0.401; $\eta_{\rho }^{2}$ = 0.031) or an age*sex interaction (*p =* 0.396; $\eta_{\rho }^{2}$ = 0.013). Thus, the young and old males and females exhibited a similar reduction in elbow flexor range of motion from the start to the end of the fatiguing exercise.

### 3.3 MVIC torque

**FIGURE 3:**
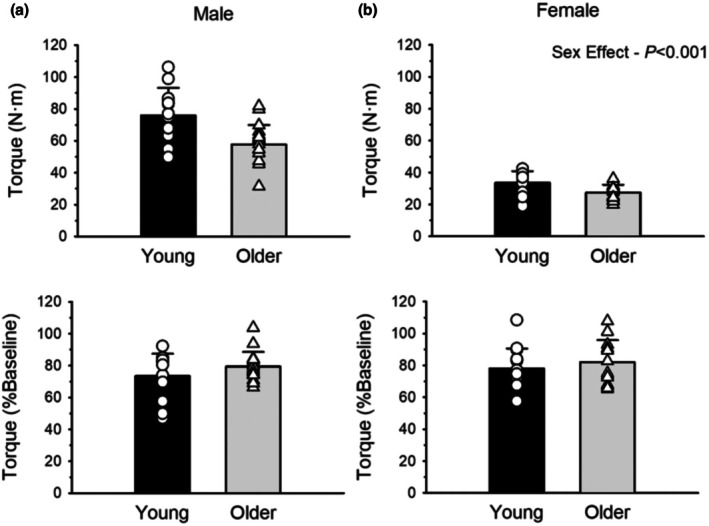
Maximal voluntary isometric contraction (MVIC) torque at baseline and immediately after dynamic, fatiguing exercise expressed relative to baseline for males (a) and females (b). Young and older adults did not differ with baseline MVIC torque (*p* = 0.060); however, males produced more baseline MVIC torque compared to females (*p* < 0.001). All groups showed a similar reduction in MVIC torque as a result of the dynamic exercise. Values are mean ± SD with individual participant data (young = open circles; older = open triangles).

Baseline MVIC torque was not different between the young (54.9 ± 25.1 N·m) and older adults (43.7 ± 17.8 N·m; age effect, *p =* 0.060, $\eta_{\rho }^{2}$ = 0.236; Figure 3). However, MVIC torque of males (66.1 ± 17.0 N·m) was over double (116%) that of the females (30.6 ± 6.7 N·m, sex effect, *p <* 0.001; $\eta_{\rho }^{2}$ = 0.733).

#### 3.3.1 Fatigability

MVIC torque declined from baseline to immediately‐post the dynamic fatiguing exercise for all groups (time effect, *p <* 0.001, $\eta_{\rho }^{2}$ = 0.762). The relative reduction in MVIC torque did not differ across all groups with no effect of age (*p =* 0.121, $\eta_{\rho }^{2}$ = 0.042; Young = 24.1 ± 13.1%; Older = 19.2 ± 11.3%), sex (*p =* 0.248, $\eta_{\rho }^{2}$ = 0.024; Males = 23.1 ± 11.6%; Females = 19.7 ± 13.0%) or an age*sex interaction (*p =* 0.758; $\eta_{\rho }^{2}$ = 0.002; Figure 3).

### 3.4 Voluntary activation

*Baseline* voluntary activation assessed from the five sets of MVIC‐60‐80% contractions and calculated with the estimated resting twitch (Equation 1) did not differ between the young (95.1 ± 3.6%) and older adults (94.4 ± 4.4%; age effect, *p =* 0.355, $\eta_{\rho }^{2}$ = 0.016; Table 3). However, there was a sex difference (sex effect, *p =* 0.002, $\eta_{\rho }^{2}$ = 0.156) in that males (96.2 ± 3.3%) had a higher voluntary activation than females (93.1 ± 4.2%). There was no age*sex interaction (*p =* 0.208; $\eta_{\rho }^{2}$ = 0.029). Note that data was not obtained from one older woman for baseline voluntary activation calculated from Equation 1.

**TABLE 3:** Baseline neuromuscular performance measures for the young and older males and females.

Variable	Young		Older	
	Male (14)	Female (14)	Male (17)	Female (15)
Electrical stimulation				
Potentiated twitch torque, N·m* ^,^ ^#^	15.1 ± 4.0	8.2 ± 2.2	11.7 ± 3.3	7.2 ± 2.1
Rate of torque development, Nm/ms* ^,^ ^#^ ^,^ ^†^	348.6 ± 107.2^b^, ^c^, ^d^	170.4 ± 45.5^a^	238.4 ± 79.4^a^, ^d^	146.2 ± 47.9^a^, ^c^
1/2 relaxation time, ms* ^,^ ^#^ ^,^ ^†^	66.1 ± 10.6^b^, ^c^, ^d^	105.5 ± 13.2^a^	94.0 ± 27.1^a^, ^d^	109.9 ± 14.6^a^, ^c^
BB M_max_ amplitude, mV* ^,^ ^#^ ^,^ ^†^	13.7 ± 5.9 (13)^b^, ^c^, ^d^	6.6 ± 3.3^a^	8.1 ± 3.6^a^	6.4 ± 4.0^a^
BB M‐wave area, mV·ms^#^	65.6 ± 32.1 (13)	34.2 ± 18.6	44.5 ± 18.2	34.7 ± 17.8
BR M_max_ amplitude, mV^#^	8.5 ± 5.6 (13)	4.8 ± 2.9 (10)	5.9 ± 3.2 (16)	4.6 ± 1.6
BR M‐wave area, mV·ms^#^	45.1 ± 35.4 (13)	23.2 ± 21.7 (10)	30.8 ± 17.8 (15)	23.4 ± 8.9
Transcranial magnetic stimulation				
Voluntary activation (Equation 1), %^#^	96.0 ± 3.7	94.2 ± 3.5	96.4 ± 3.1	92.0 ± 4.8 (14)
Voluntary activation (Equation 2), %^#^	0.51 ± 0.55	1.62 ± 1.18	0.75 ± 0.90	1.57 ± 1.29
Estimated resting twitch, N·m^#^	9.7 ± 3.5	7.4 ± 2.7	9.1 ± 4.8	5.0 ± 1.9
Normalized peak relaxation rate, s^−1^ ^#^	−8.0 ± 2.0	−6.7 ± 1.2	−8.3 ± 1.1	−5.4 ± 1.6
BB MEP_max_, %M_max_	43.9 ± 19.8 (13)	66.8 ± 31.9	54.1 ± 30.9	50.3 ± 16.6
BR MEP_max_, %M_max_	57.5 ± 38.8 (13)	49.4 ± 19.6 (10)	45.2 ± 21.8 (15)	51.3 ± 15.1

*Note*: Values are reported as mean ± SD. The sample sizes (*n*) for each cohort and certain variables are reported in parentheses.

Abbreviations: BB, biceps brachii; BR, brachioradialis; M_max_, maximal m‐wave; MEP_max_, maximal motor‐evoked potential.

^a^ Different from young males (*p <* 0.05).

^b^ Different from young females (*p <* 0.05).

^c^ Different from older males (*p <* 0.05).

^d^ Different from older females (*p <* 0.05).

* *p* < 0.05, significant effect of age.

^#^ *p <* 0.05, significant effect of sex.

^†^ *p* < 0.05, significant age × sex interaction.

After the fatiguing dynamic exercise, voluntary activation using Equation 1 was not able to be calculated in 21 of 60 participants (5 young females, 2 young males, 5 old females, and 9 old males) because of the nonlinearity of the regression to estimate peak twitch torque (i.e., the 3‐point regression *R* ^*2*^ < 0.80). As a result, the voluntary activation calculations from Equation 2 were used for post‐dynamic exercise task comparisons.

Baseline voluntary activation calculated with Equation 2 did not differ between the young (1.1 ± 1.1%) and older adults (1.1 ± 1.2%; age effect, *p =* 0.714, $\eta_{\rho }^{2}$ = 0.002). However, males (0.64 ± 0.76%) had a greater voluntary activation than females (1.6 ± 1.2%, sex effect, *p <* 0.001, $\eta_{\rho }^{2}$ = 0.194, Table 3). There was no age*sex interaction (*p =* 0.590; $\eta_{\rho }^{2}$ = 0.005).

#### 3.4.1 Fatigability

**FIGURE 4:**
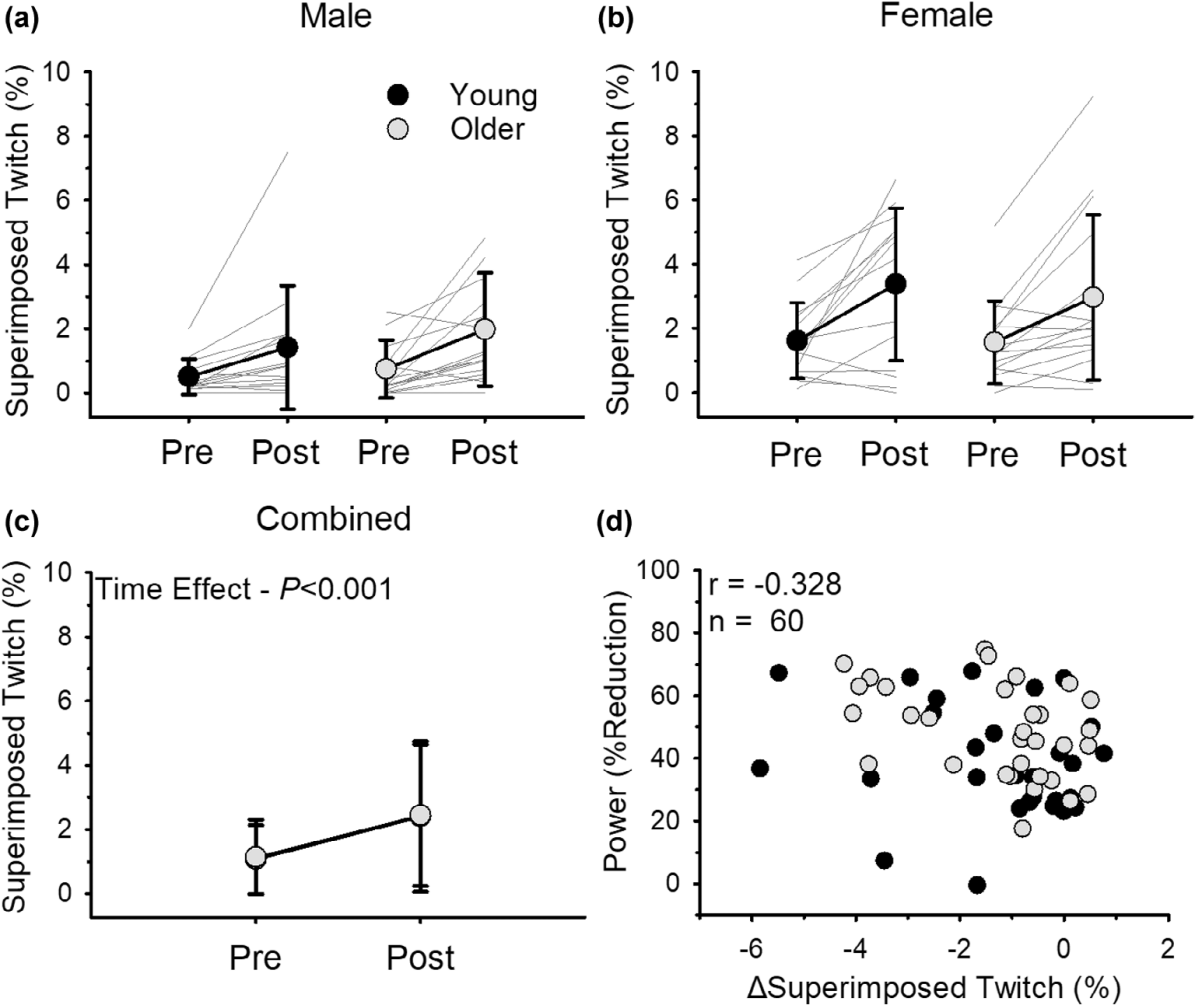
Voluntary activation (Equation 2) from the motor cortex before and immediately after dynamic exercise for males (a) and females (b). Males and females had similar reductions in voluntary activation as a result of exercise (*p* = 0.223). (c) shows combined data for males and females. Young and older adults had a similar reduction in voluntary activation as a result of the dynamic exercise (*p* = 0.957). (d) shows the association between fatigability (% reduction in power) and the reduction in voluntary activation. Fatigability was associated with the reduction in voluntary activation (Equation 2) (*r* = −0.328, *p* = 0.010, *n* = 60). Values are mean ± SD with individual participant data represented by gray lines for (a)–(c) and individual participant data are represented by dots for (d).

Voluntary activation declined following the dynamic fatiguing exercise for all groups (effect of time, *p <* 0.001, $\eta_{\rho }^{2}$ = 0.425; Figure 4) with no effect of age (*p* = 0.957, $\eta_{\rho }^{2}$ = 0.000), sex (*p* = 0.223, $\eta_{\rho }^{2}$ = 0.026), or an age*sex interaction (*p* = 0.408, $\eta_{\rho }^{2}$ = 0.012). When young and old adults were pooled, fatigability (% reduction in power) was associated with the reduction in voluntary activation (Equation 2) (*r* = −0.328, *p* = 0.010; Figure 4d) such that individuals who experienced the greatest fatigability demonstrated the largest reduction in voluntary activation.

### 3.5 M‐waves and MEPs

*Baseline* M‐wave peak‐to‐peak amplitudes (M_max_) and areas for the BB and BR are presented in Table 3. Because M_max_ and M‐wave areas behaved similarly as a result of the exercise task for both the BB and BR, only the BB results regarding responses to the exercise task are reported. The M_max_ for the BB did not change from baseline to after the fatiguing exercise across all groups (time effect, *p* = 0.845, $\eta_{\rho }^{2}$ = 0.001). Similarly, the M‐wave area for the BB did not change from baseline to after the fatiguing exercise across all groups (time effect, *p* = 0.255, $\eta_{\rho }^{2}$ = 0.024).

**FIGURE 5:**
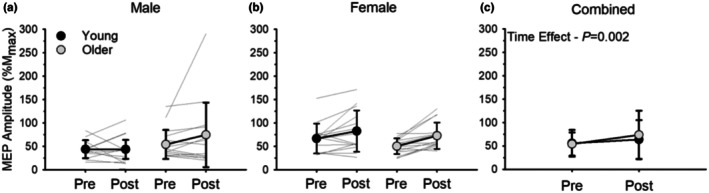
Peak‐to‐peak motor‐evoked potential (MEP) amplitude in the biceps brachii (BB) normalized to maximal M‐wave amplitude (%M_max_) before and immediately after dynamic exercise for males (a) and females (b) (*n* = 59). Females had a greater relative increase in MEP amplitude compared to males as a result of exercise (*p* = 0.042). (c) shows combined data for males and females. Both young and older adults had a similar relative increase in MEPs as a result of the dynamic exercise (*p* = 0.138). Values are mean ± SD with individual participant data represented by gray lines.

*Baseline* MEPs assessed during the MVIC for the BB and BR are presented in Table 3. Peak‐to‐peak MEP amplitude (%M_max_) for the BB increased from the start to after the dynamic fatiguing exercise (time effect, *p* = 0.002, $\eta_{\rho }^{2}$ = 0.162; Figure 5) with young and older adults showing a similar increase in MEP amplitude (age effect, *p* = 0.138, $\eta_{\rho }^{2}$ = 0.041). Females had a greater increase in MEP amplitude than males (sex effect, *p =* 0.042, $\eta_{\rho }^{2}$ = 0.076). There was no age*sex interaction (*p =* 0.640, $\eta_{\rho }^{2}$ = 0.004). Peak‐to‐peak MEP amplitude (%M_max_) for the BR did not change from baseline values to after the fatiguing exercise across all groups (time effect, *p* = 0.283, $\eta_{\rho }^{2}$ = 0.025). Note that we were unable to obtain reliable EMG data from one young and two older males for the BB. When young and older adults were pooled, there was no association between fatigability (% reduction in power) and the relative change in peak‐to‐peak MEP amplitude (%M_max_) for the BB (r = −0.189, *p* = 0.158).

### 3.6 Involuntary contractile properties from electrical stimulation

#### 3.6.1 Potentiated resting twitch (Q_tw_)

Baseline contractile properties from electrical stimulation of the biceps are presented in Table 3. Baseline Q_tw_ amplitude and rate of torque development were greater in young adults compared to older (*p* < 0.05) and greater in males compared with females (*p* < 0.05). There was an age*sex interaction (*p =* 0.021, $\eta_{\rho }^{2}$ = 0.090) with younger males having a greater Q_tw_ rate of torque development than older males (*p* < 0.001), but no differences between young and older females (*p* = 0.384). Baseline Q_tw_ half relaxation time was less in the young adults than older adults (*p* = 0.001, $\eta_{\rho }^{2}$ = 0.173) and in the males compared with the females (*p* < 0.001, $\eta_{\rho }^{2}$ = 0.382). Additionally, there was an age*sex interaction (*p =* 0.015, $\eta_{\rho }^{2}$ = 0.101) with younger males having a shorter Q_tw_ half relaxation time than older males (*p* < 0.001), but no differences between young and older females (*p* = 0.523).

#### 3.6.2 Fatigability

**FIGURE 6:**
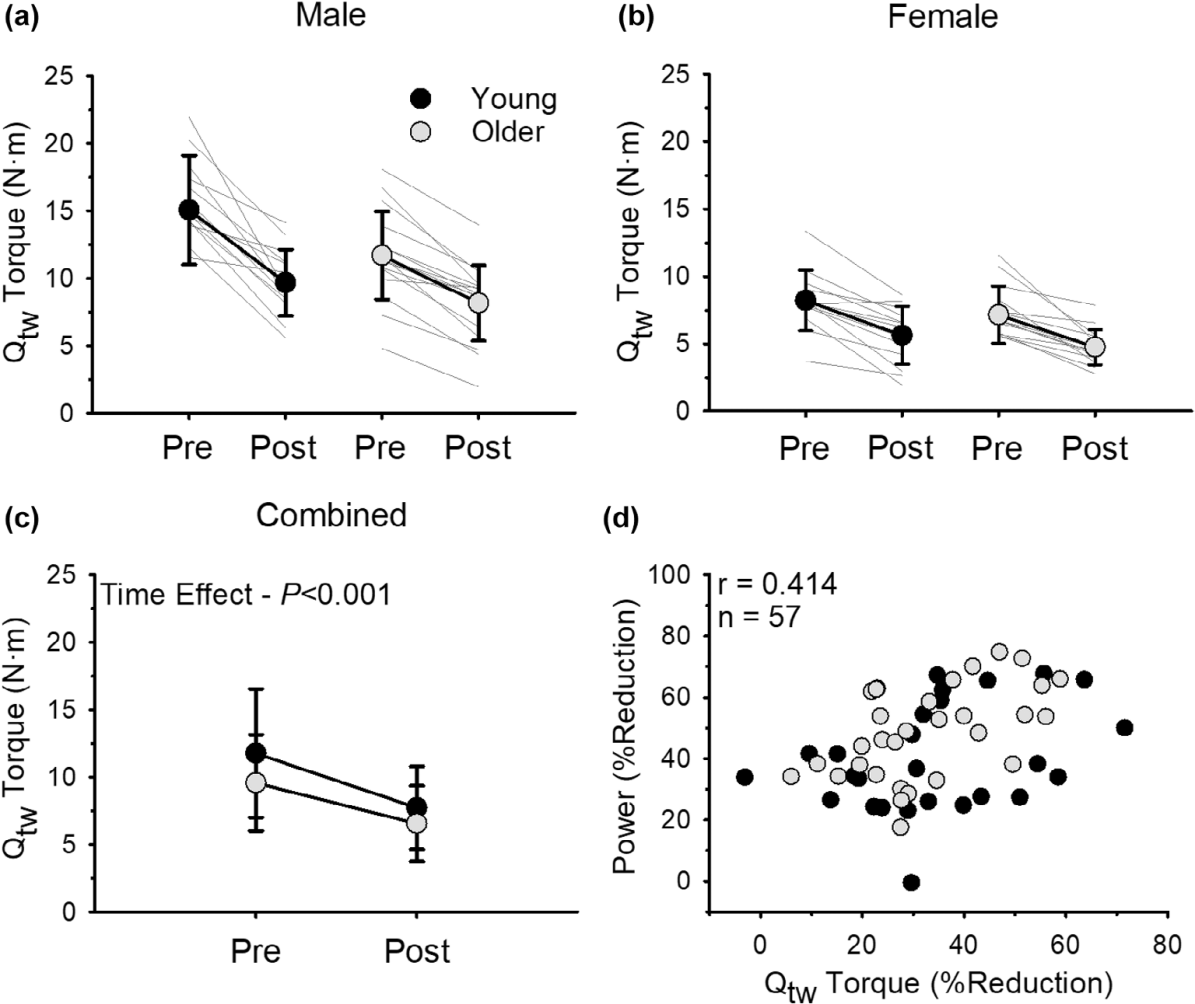
Potentiated resting twitch (Q_tw_) amplitude before and immediately after dynamic exercise for males (a) and females (b) (*n* = 57). Males and females had similar reductions in Q_tw_ amplitude as a result of exercise (*p* = 0.765). (c) shows combined data for males and females. Both young and older adults had a similar reduction in Q_tw_ amplitude as a result of the dynamic exercise (*p* = 0.719). (d) shows the association between fatigability (% reduction in power) and the relative reduction in Q_tw_ amplitude. Fatigability was associated with the relative reduction in Q_tw_ amplitude (*r* = 0.414, *p* < 0.001, *n* = 57). Values are mean ± SD with individual participant data represented by gray lines for (a)–(c) and individual participant data are represented by dots for (d).

The relative (%) reduction in the Q_tw_ amplitude at the end of the fatiguing exercise did not differ across all groups with no age (*p =* 0.719, $\eta_{\rho }^{2}$ = 0.002; Young = −34.3 ± 17.7%; Older = −32.6 ± 13.9%) or sex differences (*p =* 0.765, $\eta_{\rho }^{2}$ = 0.002; Males = −33.8 ± 14.8%; Females = −32.9 ± 16.7%; Figure 6). Similarly, the relative reduction in the Q_tw_ rate of torque development did not differ across all groups with no age (*p =* 0.446, $\eta_{\rho }^{2}$ = 0.011; Young = −36.3 ± 23.3%; Older = −40.4 ± 17.3%) or sex differences (*p =* 0.588, $\eta_{\rho }^{2}$ = 0.005; Males = −39.9 ± 20.8%; Females = −37.0 ± 19.7%). The relative increase in the Q_tw_ half relaxation time did not differ across all groups with no age (*p =* 0.744, $\eta_{\rho }^{2}$ = 0.002; Young = 49.6 ± 50.2%; Older = 54.5 ± 56.0%) or sex differences (*p =* 0.125, $\eta_{\rho }^{2}$ = 0.042; Males = 62.6 ± 57.8%; Females = 40.8 ± 45.6%). Q_tw_ amplitude data from three participants (one young female, one young male, and one older female) was not included in the analysis due to issues with the measurement during the post‐exercise contractile property assessments.

When young and old adults were pooled together, there was an association between fatigability (% reduction in power) and the relative reduction in Q_tw_ amplitude (*r* = 0.414, *p* < 0.001; Figure 6d), the relative reduction in Q_tw_ rate of torque development (*r* = 0.316, *p* = 0.015), and the relative increase in the Q_tw_ half relaxation time (*r* = 0.458, *p* < 0.001). Thus, individuals who experienced the greatest fatigability also demonstrated a greater loss in peak twitch torque and slowing of the torque development and relaxation of the muscle.

### 3.7 Peak rates of relaxation from TMS

*Baseline* normalized peak rates of torque relaxation (s^−1^) of the elbow flexor muscles elicited by TMS during the MVICs is presented in Table 2. Normalized peak relaxation rates did not differ between young and older adults (*p* = 0.208, $\eta_{\rho }^{2}$ = 0.028), but were greater in males compared with females (*p* < 0.001, $\eta_{\rho }^{2}$ = 0.334).

#### 3.7.1 Fatigability

**FIGURE 7:**
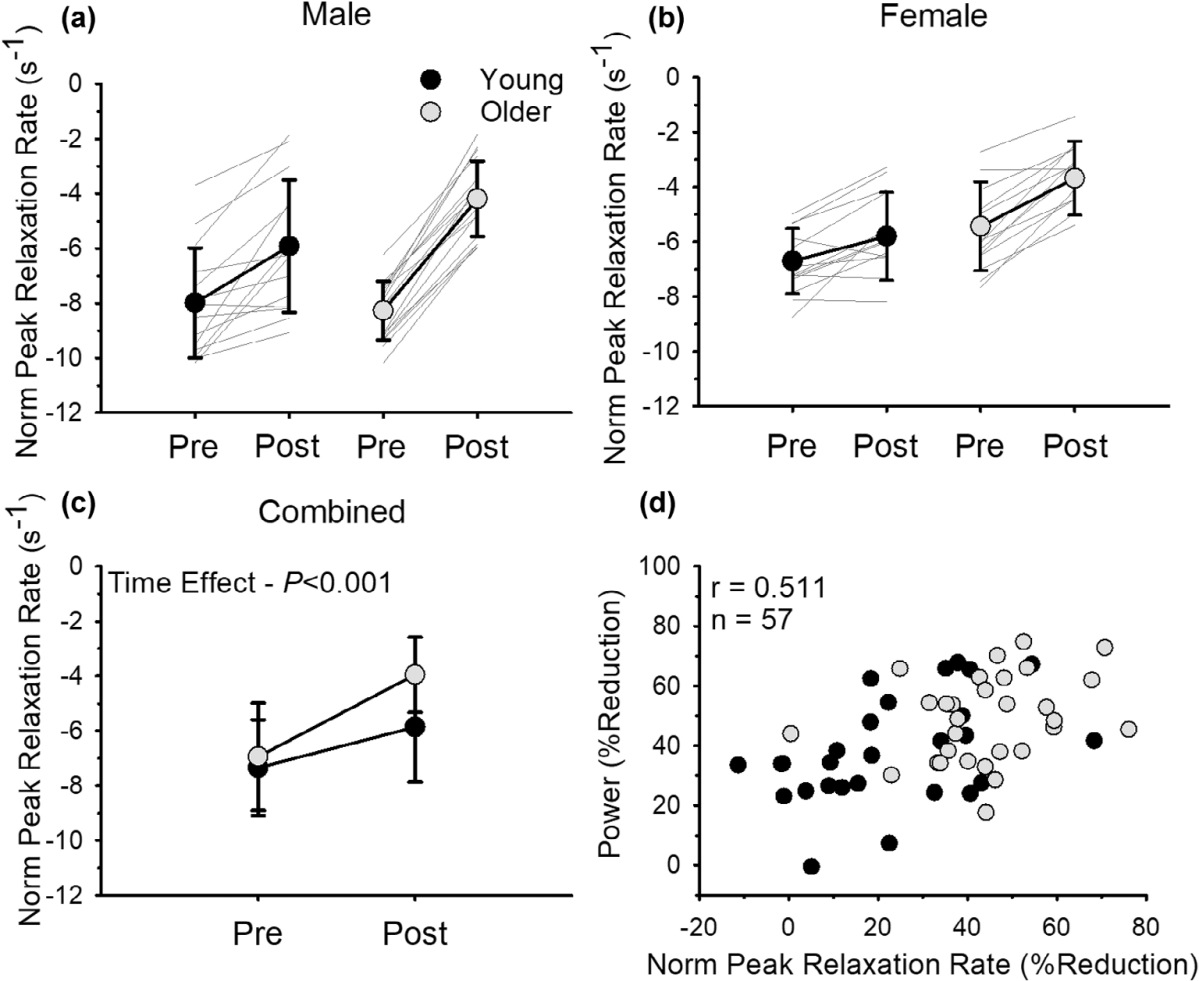
Normalized (s−1) peak rates of torque relaxation before and immediately after dynamic exercise for males (a) and females (b) (*n* = 57). Males had a greater relative reduction in relaxation rates compared to females (*p* = 0.013). (c) shows combined data for males and females. Older adults had a greater reduction in relaxation rates compared to young (*p* < 0.001). (d) shows the association between fatigability (% reduction in power) and the relative reduction in normalized peak rates of torque relaxation. Fatigability was associated with the relative reduction in normalized peak rates of torque relaxation (*r* = 0.511, *p* < 0.001, *n* = 57). Values are mean ± SD with individual participant data represented by gray lines for (a)–(c) and individual participant data are represented by dots for (d).

The relative decrease in the normalized peak rate of torque relaxation was greater in older adults (44.4 ± 15.1%) compared with young adults (22.8 ± 19.2%; age effect, *p <* 0.001, $\eta_{\rho }^{2}$ = 0.299; Figure 7) and greater in males (39.8 ± 20.4%) compared with females (27.5 ± 18.1%; sex effect, *p =* 0.013, $\eta_{\rho }^{2}$ = 0.110). These data did not include three outliers (one young female; two older females) whose data were removed. When young and old adults were pooled together there was an association between fatigability (% reduction in power) and the relative reduction in normalized peak rates of torque relaxation (*r* = 0.511, *p* < 0.001; Figure 7d). Thus, individuals who experienced the greatest fatigability also demonstrated a greater slowing of the relaxation of the muscle measured from the superimposed twitch elicited by TMS.

## 4 DISCUSSION

The purpose of this study was to determine the mechanisms for the age‐related increase in fatigability during high‐velocity contractions of the elbow flexor muscles. We found that the elbow flexor muscles of the older adults were more fatigable than young adults (~11% age difference), and the mechanisms were primarily of muscular origin. These findings were reflected in the greater reduction in involuntary normalized peak rates of torque relaxation in older adults than young adults that was associated with the reduction in power during the fatiguing exercise. In contrast to our hypothesis, we did not observe any sex differences in the age‐related increase in fatigability of the elbow flexors. Furthermore, while there were significant associations between the reduction in voluntary activation and the reduction in power during the fatiguing exercise in both young and older adults, the associations were weak. There were also no age differences in the reductions in voluntary activation, suggesting that impaired neural activation was not contributing to the age‐related increase in fatigability. These results largely parallel the findings from the lower limb muscles (Sundberg, Kuplic, et al., 2018) and suggest that the mechanisms for the age‐related increase in fatigability in the elbow flexor muscles are primarily within the muscle.

### 4.1 Age‐related fatigability and absolute power output of the elbow flexors muscles

Our findings of an ~11% greater reduction in power of the elbow flexor muscles in older adults than young adults are similar to that previously reported (Senefeld et al., 2017) and less than that reported in the knee extensor muscles (Paris et al., 2022; Senefeld et al., 2017; Sundberg, Kuplic, et al., 2018). It could be that these limb differences are driven by varying use patterns between the upper and lower limbs with aging. One study identified that the elbow flexor muscles of young adults were activated nearly twice as much throughout the day when compared to the knee extensor muscles (Kern et al., 2001). Indeed, it is thought that the varying use patterns contribute to the variation in age‐related muscular atrophy and strength between the upper and lower limb (Frontera et al., 2000; Naruse et al., 2023) and could contribute to the reduced age‐related increase in fatigability of the upper limb compared to lower limb muscles. Additionally, aging has been shown to have differential effects on exercise hyperemic responses between the upper and lower limb, with exercise hyperemia being preserved in the upper limb but attenuated in the lower limb compared with young adults (Donato et al., 2006; Wray et al., 2010). Furthermore, a preserved oxidative capacity has been observed in the upper limb muscles of older adults, whereas knee extensor oxidative capacity has been shown to be attenuated in older adults compared to young adults (Fitzgerald et al., 2016), although this is not always observed and is a topic of an ongoing debate (Lanza et al., 2025; Marcinek & Ferrucci, 2025). Both a preserved exercise hyperemia and oxidative capacity in the upper limb with aging could attenuate the age‐related increase in fatigability in the upper limb.

Absolute power output at the beginning of the dynamic exercise task was 40% higher in young adults compared with older adults. These results differ from age‐related differences in power output observed in the knee extensor muscles at a similar percentage of MVIC (20%) with young adults having a 97% higher power output than older adults (Sundberg, Kuplic, et al., 2018). The dissimilarity seen in age‐related differences in power output between the upper and lower limb is likely due to the varying use patterns between the limbs leading to a greater preservation of upper limb power output with age (Frontera et al., 2000; Janssen et al., 2000; Kern et al., 2001; Naruse et al., 2023).

### 4.2 Contractile mechanisms contribute to greater age‐related fatigability of the elbow flexors

A novel finding of this study is that the greater fatigability of the elbow flexor muscles in older adults is driven primarily by contractile mechanisms. We found a ~22% greater relative reduction in the peak rates of torque relaxation in older compared with young adults that was associated with the reduction in power during the fatiguing exercise. Furthermore, other indices of slowing of the muscle, including a prolongation of the half‐relaxation time and a reduction in the Q_tw_ rate of torque development, were observed in both groups and associated with the reduction in power. The contractile origin for the age‐related increase in fatigability of the elbow flexors is largely similar to that found in the knee extensor muscles during dynamic contractions (Dalton et al., 2012; Sundberg, Kuplic, et al., 2018). For example, Dalton et al. (2012) observed a greater reduction in knee extension power in older adult males compared with young in response to fast‐velocity isotonic contractions that was associated with a slowing of the muscle, as identified through prolongation of the half‐relaxation time. Additionally, it was found that the greater knee extensor fatigability in older males and females during high‐velocity contractions was associated with reductions in the involuntary rates of torque development, prolongation of the half‐relaxation time, and reductions in the normalized peak rate of torque relaxation (Sundberg, Kuplic, et al., 2018). The consistency of the findings between muscle groups suggests that mechanisms eliciting slowed calcium (Ca^2+^) uptake into the sarcoplasmic reticulum and/or prolonged cross‐bridge attachment times could play a role in the age‐related increased fatigability.

The cellular and molecular mechanisms leading to the slowed relaxation of the muscle are thought to be primarily driven by the accumulation of hydrogen ions (H^+^) during fatiguing contractions (Debold et al., 2016; Sundberg & Fitts, 2019). Elevated H^+^ has been shown to increase the time of myosin's attachment to actin as a result of a slowed ADP release from myosin (Debold et al., 2008), as well as slow the reuptake of Ca^2+^ back into the sarcoplasmic reticulum (Allen et al., 1995). This in conjunction with recent findings that a greater accumulation of H^+^ in older adults compared with young during dynamic knee extension exercise was closely associated with the reduction in power (Sundberg et al., 2019) supports the potential role of elevated H^+^ in the current findings in the elbow flexor muscles.

In addition to the slowed relaxation of the muscle, we observed a marked reduction in the Q_tw_ amplitude for all groups that was associated with the reduction in power during the fatiguing task. This finding is supported by similar findings of the knee extensor muscles (Sundberg, Hunter, et al., 2018; Sundberg, Kuplic, et al., 2018) and is largely thought to be due to the elevated levels of both H^+^ and inorganic phosphate (P_i_) (Debold et al., 2016; Sundberg & Fitts, 2019). Specifically, elevated P_i_ and H^+^ directly inhibit force and power of the cross‐bridge in humans (Foster et al., 2025; Sundberg et al., 2025; Sundberg, Hunter, et al., 2018) and decrease myofibrillar Ca^2+^ sensitivity, whereas P_i_ also inhibits Ca^2+^ release from the sarcoplasmic reticulum (Debold et al., 2016; Fryer et al., 1995; Sundberg & Fitts, 2019). Although a marked reduction in the Q_tw_ amplitude was identified and provides insights into the mechanisms for the fatigability of the elbow flexor muscles irrespective of age or sex, we did not observe an age difference in the reduction in the Q_tw_ amplitude, and future studies are needed to identify the mechanisms within the elbow flexor muscles.

### 4.3 Voluntary activation and corticospinal excitability

Neural drive from the motor cortex decreased in both cohorts immediately following the fatiguing exercise and was associated with the reduction in power. This finding suggests a reduced neural drive contributed to the fatigability of the elbow flexor muscles for high‐velocity, dynamic contractions for both young and older adults, but did not explain the age‐related differences in fatigability. Results from other studies are mixed regarding neural drive following high‐velocity, dynamic contractions, with some studies showing no change in neural drive (Dalton et al., 2010; Fitzgerald et al., 2020; Sundberg, Kuplic, et al., 2018) and others supporting our current findings of a reduced neural drive (Dalton et al., 2012, 2015). Regardless, the reductions in voluntary activation were not large in either age group and could reflect increased inhibitory feedback from group III and IV afferent fibers that interfered with the net excitability of the motor neuron pool (Gandevia, 2001; Hunter, 2018; Hureau et al., 2022).

To evaluate the excitability of the sarcolemma and propagation at the neuromuscular junction, we measured the M‐wave responses of the BB and BR and found no change in either the M‐wave amplitude or area after the fatiguing exercise for either age group. Although changes within neuromuscular junction stability have been suggested with aging (Hepple & Rice, 2016; Hunter et al., 2016), our results suggest that age‐related alterations at the neuromuscular junction did not play a major role in the fatigability of the elbow flexor muscles in either young or older adults. These results are consistent with our previous studies and others showing no change in the M‐wave during isometric and slow‐velocity, dynamic fatiguing exercise in the elbow flexors in young and older adults (Yoon et al., 2012, 2015).

In contrast to the lack of a change in the excitability of the sarcolemma, the MEP amplitude elicited by TMS increased in the BB after the fatiguing exercise, indicating an increased excitability of the corticospinal tract across all groups. However, the increase in MEP amplitude of the BB was similar for all groups and was not associated with the reduction in power. An increase in cortical excitability has also been observed previously in the elbow flexor muscles during isometric (Hunter et al., 2008; Klass et al., 2008; Sogaard et al., 2006) and slow‐velocity dynamic exercise (Yoon et al., 2015), although not always (Marzouk et al., 2023; Yoon et al., 2012). This increased corticospinal excitability is thought to in part compensate for impaired contractile function in the muscle and decreased spinal excitability during exercise, and is often accompanied by increased effort (Taylor et al., 2016).

### 4.4 Sex‐related differences in power and fatigability

At baseline, absolute power output at the beginning of the dynamic exercise task was 127% higher in males than females and is larger than the 64%–68% sex differences observed in the knee extensor muscles (Sundberg, Kuplic, et al., 2018; Wrucke et al., 2024). The greater sex difference in power output for the upper limb was similar across both age groups and likely due to the larger sex difference in muscle mass and distribution of type II muscle fibers in the upper limb compared to the lower limb (Hunter et al., 2023; Nuzzo, 2023, 2024). Additionally, we found no differences between males and females in the magnitude of age‐related fatigability of the elbow flexors for high‐velocity contractions. While this finding is similar to that of the knee extensor muscles for a similar task (Sundberg, Kuplic, et al., 2018), it is in contrast to our previous findings showing a greater fatigability of the elbow flexor muscles in older males compared to females (Senefeld et al., 2017). The conflicting findings could be due to differences in the fatiguing task. Senefeld et al. (2017) employed a similar high‐velocity fatiguing exercise task at the same MVIC load (20%), but differed from the current protocol by including three sets of 30 dynamic contractions with an MVIC performed immediately after each set and a brief rest period of ~6 s before starting the next set. With females having enhanced recovery following dynamic contractions (Senefeld et al., 2018), the brief period of rest could have contributed to the lesser fatigability in older females. While the different fatiguing protocols may have contributed to the variation in the magnitude of sex differences in fatigability, the age‐related increase in fatigability was consistent across both fatiguing tasks. Lastly, we show a greater reduction (~13%) in the normalized peak rates of torque relaxation in males compared to females. This finding is similar to that observed in the elbow flexor muscles during a moderate velocity (60°/s) fatiguing task (Yoon et al., 2015). However, this greater impairment in muscle relaxation in males did not lead to sex differences in age‐related fatigability.

## 5 CONCLUSION

This study provides mechanistic insight into the age‐related increase in fatigability of the elbow flexor muscles for high‐velocity, dynamic contractions. Contractile mechanisms contributed to the age‐related increase in fatigability, as seen in the greater reduction in rates of relaxation following the dynamic fatiguing exercise and the associations with fatigability. Other indices of contractile impairment (e.g., Q_tw_ amplitude) were also associated with fatigability but did not differ between young and older adults, nor between the sexes. Lastly, neural mechanisms contributed to the fatigability of the elbow flexor muscles in young and older adults but did not explain the age‐related increase in fatigability. These results suggest cellular mechanisms that contribute to altered contractile function of the muscle play a primary role in the age‐related increase in fatigability of the elbow flexor muscles, similarly in males and females.

## AUTHOR CONTRIBUTIONS

C.W.S. and S.K.H. conceived and designed research; A.K., M.D.A, and C.W.S. performed experiments; B.E.A., A.K., M.D.A., and C.W.S. analyzed data; B.E.A., C.W.S., and S.K.H. interpreted results of experiments; B.E.A. prepared figures; B.E.A., A.K., and S.K.H. drafted manuscript; B.E.A, A.K., M.D.A, C.W.S., and S.K.H. edited and revised manuscript; B.E.A, A.K., M.D.A, C.W.S., and S.K.H. approved final version of manuscript.

## FUNDING INFORMATION

This work was supported by the National Center for Advancing Translational Sciences, National Institutes of Health award (2TL1R001437) to B. E. Arney and a National Institute of Aging R01 grant (AG048262) to S. K. Hunter, R. H. Fitts, and C. W. Sundberg.

## CONFLICT OF INTEREST STATEMENT

No conflicts of interest, financial or otherwise, are declared by the authors. Contents from this manuscript were reused/adapted from Kuplic (Kuplic, 2017).

## Data Availability

Data will be made available upon reasonable request.
